# Clinical investigation plan for the use of interactive binocular treatment (I-BiT) for the management of anisometropic, strabismic and mixed amblyopia in children aged 3.5–12 years: a randomised controlled trial

**DOI:** 10.1186/s13063-019-3523-0

**Published:** 2019-07-16

**Authors:** Rebecca Brown, Peter Blanchfield, Apostolos Fakis, Paul McGraw, Alexander J. E. Foss, And on behalf of the I-BiT Study Group

**Affiliations:** Department of Ophthalmology, Queen’s Medical Centre, Nottingham, NG7 2UH UK

**Keywords:** Amblyopia, I-BiT™, Randomised clinical trial, Lazy eye, Child

## Abstract

**Background:**

Amblyopia (lazy eye) affects the vision of approximately 2% of all children. Traditional treatment consists of wearing a patch over their ‘good’ eye for a number of hours daily, over several months. This treatment is unpopular and compliance is often low. Therefore, results can be poor. I-BiT is a system, based on stereo technology using shutter glasses, designed to treat amblyopia using dichoptic stimulation. This trial uses a redesigned system for home use and includes eye-tracking capability.

**Methods/design:**

This is a randomised controlled trial involving three groups of 40 patients each, aged between 3.5 and 12 years, with a diagnosis of (1) anisometropic amblyopia, (2) mixed or strabismic amblyopia prior to strabismic surgery and (3) mixed or strabismic amblyopia who have just undergone strabismus surgery. They will be randomised in a 1:1 ratio between I-BiT and control and will receive treatment, at home over a 6-week period. Their visual acuity will be assessed independently at baseline, mid-treatment (week 3), at the end of treatment (week 6) and, for those receiving the active I-BiT treatment, 4 weeks after completing treatment (week 10).

The primary endpoint will be the change in visual acuity from baseline to the end of treatment. Secondary endpoints will be additional visual acuity measures, patient acceptability, compliance and the incidence of adverse events.

**Discussion:**

This is a randomised controlled trial using the redesigned I-BiT™ system to determine if this is a feasible treatment strategy for the management of anisometropic, strabismic and mixed amblyopia.

**Trial registration:**

ISRCTN Number/Clinical trials.gov, ID: NCT02810847. Registered on 23 June 2016.

**Electronic supplementary material:**

The online version of this article (10.1186/s13063-019-3523-0) contains supplementary material, which is available to authorized users.

## Background

The term amblyopia is derived from the Greek, meaning blunted sight and is defined as a unilateral or bilateral decrease in vision for which no obvious cause can be found on clinical examination, or as von Graefe phrased it, a condition where the observer sees nothing and the patient very little [1]. It is a common condition affecting 2–3% of the population [2].

Amblyopia is characterised by:-

- Reduced visual acuity
- The phenomenon of crowding
- Presence of a central suppression scotoma
- Impairment of binocular vision

Von Graefe was the first to demonstrate suppression in a lazy eye [3] and there is evidence of suppression in all amblyopes [4].

The first-line treatment is the correction of any existing significant refractive error for at least 18 weeks (refractive adaptation) but full resolution only occurs in about 30% [5, 6]. For patients in whom the amblyopia persists, then the mainstay of treatment has been patching, or more recently, penalisation. The major barrier to patching is that wearing patches is uncomfortable and accordingly compliance is variable. Objective measurements suggests that average concordance with patching was 48% [7]. A major factor may well be the association of increased reports of bullying by children who wear patches [8].

The second issue is that the normal eye is covered up. Accordingly, while it may improve the vision in the amblyopic eye, it can lead to reduced vision in the patched eye and there is still the outstanding issue of developing stereopsis.

An alternative treatment is to use a strategy based on dichoptic stimulation [9]. Dichoptic stimulation is the ability to present different images to each eye independently. For example, one could present the sprite (a sprite is a computer graphic which may be moved on-screen and otherwise manipulated as a single entity and here refers to a gaming object of interest) to one eye and objects to collect to the other eye thereby forcing both eyes to work together in order to successfully play the game. The I-BiT group, who were one of the first to adopt this approach, did so using a variation on the game Nux.

There is nothing original in the idea and a number of devices had been developed, starting with Priestly-Smith’s fusion tubes followed by Worth’s amblyoscope and then by the 1930s Maddox developed the major amblyoscope, or synoptophore. The synoptophore allows such tasks as putting the lion (seen by one eye) in the cage (seen by the other) and was developed very much with the idea of dichoptic stimulation for therapeutic benefit. However, such tasks were too simple to retain anyone’s interest beyond a few minutes and way short of the hours needed for any therapeutic effect.

What has changed is the rapid advances in stereo-viewing technology that has given this approach a new lease of life as it can be readily adapted for dichoptic stimulation. These considerations led us to the initial development of a virtual reality-based system [10] to treat amblyopia using dichoptic stimulation, either playing special video games or watching DVDs which we called I-BiT (*I*nteractive *Bi*nocular *T*reatment). Three pilot studies [11–13] have shown that the I-BiT™ system can improve the visual acuity in amblyopic patients. The most recent of these pilot studies using shutter-glasses technology [13] showed that all patients who completed their planned treatment (nine of the 10 patients) showed a mean improvement in visual acuity of 0.18 LogMAR. This system went to a randomised control trial (RCT) and while all the arms showed a visual improvement of 0.07 LogMAR units, there was no difference between the arms including the control (playing the videogame under non-dichoptic conditions) [14].

A group from Iran developed their own system, which they also called I-BiT but was developed independently of the Nottingham team, using dichoptic games (Pacman, Tetris and Snake) with red-green anaglyph glasses, to treat 50 patients aged 3–10 years old and again showed improvement in vision in both the I-BiT and the control arms [15] but both arms also received patching making the results hard to interpret.

The I-BiT™ system was designed for treating patients under supervision in a hospital-based setting and this significantly limits treatment times to only a few hours (3 h in the trial). There was also no attempt to ‘balance’ the images. The extra visual features that were shown to the amblyopic eye were ‘all or none’.

We have redesigned our I-BiT system for home use along with incorporation of an eye-tracker. The innovation is the combination of the two technologies of virtual reality and eye-tracking. This allows one to achieve the dual aims of dichoptic stimulation and harmonious retinal presentation with engaging visual material that will retain a child’s attention for prolonged periods of time. This combination is currently untested.

This study will aim to evaluate the new system in participants aged 3.5 to 12 years with anisometropic, mixed and strabismic amblyopia.

## Methods/design

### Objectives of the study

The trial is a feasibility study (similar to a stage 2 CTIMP trial) with the aim to show that this treatment strategy is feasible (equipment can be delivered and retrieved from patients’ homes and will function in a home setting) and to get some indication of clinical efficacy as to whether I-BiT combined with refractive correction is superior to refractive correction alone in the treatment of amblyopia in three scenarios (untreated anisometropic amblyopia, mixed or strabismic amblyopia and residual amblyopia in patients undergoing strabismus surgery).

### I-BiT™ system

The redesigned I-BiT system consists of desktop personal computer with a core 5i processor and graphics card and a 27″ HD screen. Attached to the inferior border of the screen is an eye-tracker bar with an infra-red light source. Dichoptic stimulation is achieved by using radio-wave controlled 3D active shutter glasses and a USB controller/infra-red emitter. The infra-red signal is converted to a radiofrequency signal (2.48 GigaHz) in a box which is opaque to infrared and transparent to radio waves. The active shutter glasses are to be worn by the patient as this is the means by which different images are presented to each eye. The controller/radiofrequency emitter synchronizes the glasses with the monitor so that each frame is displayed to the correct eye.

There is a games controller by which the options are selected and the games are played.

The system has the ability to perform simple psychophysical measurements (visual acuity, cover test and test for double vision). The visual acuity is measured using an illiterate E test. The cover test uses the eye-tracker and the target is shown first to the good eye, then to both and then to the amblyopic eye and the movement of the eyes is recorded when the target is switched from appearing to both eyes and is only shown to the amblyopic eye only. The test for double vision is when a bunny rabbit is shown to both eyes and the child is asked to select whether they see one rabbit or two.

It has the ability to off-set the images so as to ensure harmonious retinal presentation of images for patients with strabismic or mixed amblyopia.

### Video stimulus

I-BiT has episodes from popular young children’s programmes (with permission from the British Broadcasting Cooperation). For three of these programmes, there is a blurred patch that is presented to the central vision of the good eye and centrality is maintained by eye-tracking. For the fourth programme and software is used to detect the presence of faces in the images and then the faces that are shown to the good eye are specifically blurred but not for the amblyopic eye. The rationale is that faces have high salience.

### Games stimulus

Eight games have been programmed and they are designed to address different aspects of amblyopia:

- Two shooter games which have an anti-crowding strategy (based on Nux and Space Invaders)
- A chaser game which has an anti-suppression strategy
- A game based on Pac-Man which also has an anti-suppression strategy
- Two games that require accurate foveation of the eye (one is based on the arcade game Whack-A-Mole)
- Two games designed to stimulate binocularity and stereopsis

### Trial design

The trial protocol is version 1.4 and dated 19 December 2016. The trial is a single-masked, randomised control trial. It is not possible to mask the intervention as the participant has only to shut one eye to know whether they are in the treatment or the control arm.

The trial is split into three groups each of these three groups will investigate a distinct (and mutually exclusive) patient population which together cover most cases of anisometropic or strabismic amblyopia that presents to the National Health Service:

1. Group 1: untreated anisometropic amblyopia.
2. Group 2: strabismic or mixed amblyopia with no previous strabismus surgery (but may have had previous amblyopia treatment)
3. Group 3: immediate post-strabismus surgery. Participants with residual strabismic or mixed amblyopia undergoing alignment surgery. These participants will receive I-BiT Plus treatment starting in the immediate post-operative period

The trial will be assessor masked (it is not possible to mask the intervention) randomised intervention with 40 participants in each group with either continued refractive adaptation (if glasses are indicated and prescribed) or observation as the controls. The participants in the control arm will also be provided with a gaming computer on which they can play games and watch DVDs but will not have the capability to provide dichoptic stimulation nor provide eye-tracking. Carers will be provided with a contact number for enquiries and technical help.

In all three groups, the participants will be randomised to either receive I-BiT Plus for 6 weeks or extended refractive adaptation/observation and will be examined at weeks 3 and 6 post receipt of I-BiT Plus or laptop for both arms, and at week 10 in the I-BiT treatment arm only. In all three groups, after week 6, the participants in the control arms will be returned to standard NHS care while the intervention arms will be observed for a further 4 weeks. The rationale is that the week-6 visit is the comparison between treated and controls and is the primary endpoint. The point of the week 10 visit is to see if any improvement observed in the treated arm is stable or not and the comparison is, therefore, between the week-6 and the week-10 visits for the treated arms.

The choice of untreated controls was chosen as the most suitable choice for determining whether I-BiT is an effective treatment and superior to no treatment.

**Fig. 1:**
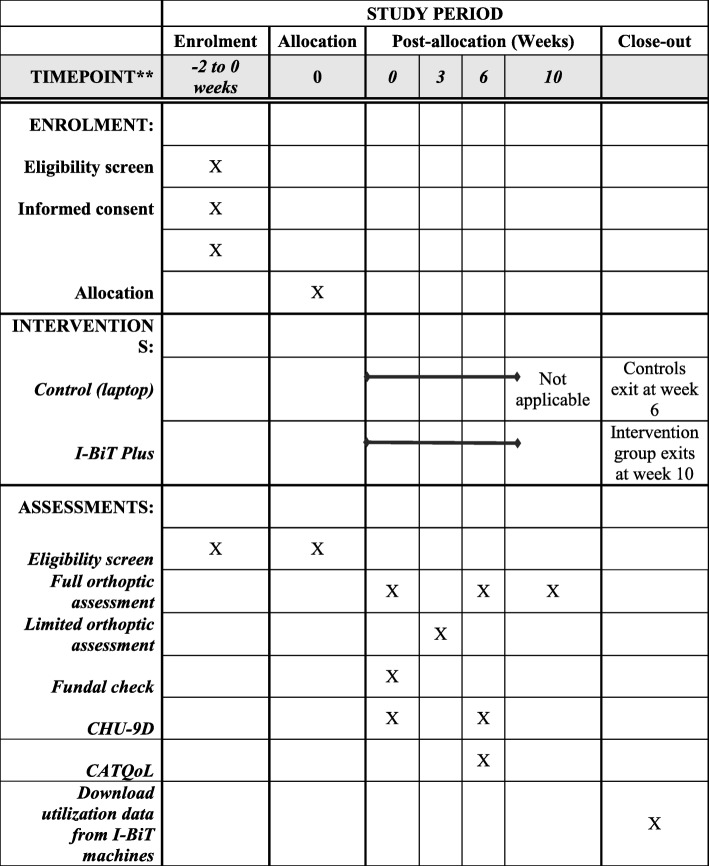
Summary of trial design

The basic study design is the same for all three groups and is summarised in Fig. 1.

### Eligibility criteria

These are summarised in Table 1.

**Table 1:** Table of inclusion and exclusion criteria for the three groups in the clinical trial

Group 1	Group 2	Group 3
Anisometropic amblyopia	Strabismic or mixed amblyopia	Post-strabismus surgery
Inclusion criteria		
3.5^5^–12^4^ years	3.5^5^–12^4^ years	3.5^5^–12^4^
Vision in amblyopic eye between 0.3 and 1.3 logMAR with an intraocular difference of 0.2 logMAR or more	Vision in amblyopic eye between 0.3 and 1.3 logMAR with an intraocular difference of 0.2 logMAR or more	Vision in amblyopic eye between 0.3 and 1.3 logMAR with an intraocular difference of 0.2 logMAR or more
May have a manifest squint of less than or equal to 10 prism dioptres	Manifest squint must be greater than 10 prism dioptres	Residual squint following surgery (no more than 4 weeks prior) of any size
Undergone refractive adaptation^1^	Undergone refractive adaptation (if indicated)^1,2^	Undergone refractive adaptation (if indicated) ^1,2^
No previous patching or penalisation	Previous patching or penalisation acceptable	Previous patching or penalisation acceptable
No previous strabismus surgery	No previous strabismus surgery	Treatment to start within 4 weeks of strabismus surgery but no previous strabismus surgery
Can use I-BiT Plus	Can use I-BiT Plus	Can use I-BiT Plus
Exclusion criteria		
Deprivational amblyopia		
Presence of manifest nystagmus		
Other ocular or neurological disease affecting the visual system^3^		
Parent, guardian or child not prepared to give consent		

^1^Refractive adaptation is wearing refractive correction for a minimum of 12 weeks

^2^Refractive adaptation is indicated if there is a significant refractive error

^3^These include Down’s syndrome, developmental delay, craniofacial syndrome, foetal alcohol syndrome and cerebral palsy among other conditions

^4^The upper limit of the age range is 1 day before their 13th birthday

^5^The lower limit of the age range is 182 days after their third birthday

### Trial settings

There are four trial centres involving the orthoptic departments of the Queen’s Medical Centre at Nottingham, Southampton General Hospital, The Royal Stoke University Hospital and Moorfields Eye Hospital in London. Patients will be recruited from these centres but the treatment itself is home based.

### Study procedures

#### Screening and eligibility assessments

Potential participants are identified from patients attending the orthoptic departments of participating centres. Aids to recruitment including displaying of posters in these departments and there is a website describing the project that is publically accessible.

### Consent

Consent is taken by the research orthoptists at the trial sites from the participant’s parents or guardians. The opportunity is given also to each participant to provide their assent. Separate parent (Additional file 1) and participant (Additional file 5) information sheets are provided and separate consent (Additional file 2) and assent  (Additional files 3 and 6) forms.

### Baseline assessments

A full orthoptic assessment is performed at baseline and will include:

- Refractive error
- Visual acuity
- Ocular motility and convergence status
- Prism Cover Test for horizontal and vertical deviation for near and distance
- Cover test
- Binocularity status (including stereoacuity)
- Fundal check (if not recorded in the hospital record)
- Quality of life measurement using the CHU-9D.

Visual acuity will be measured by one of Keeler LogMAR crowded, crowded LogMAR Kays pictures or the Bailey-Lovie LogMAR test depending upon the child and the same test will be used throughout for each participant. The amblyopic eye is always tested first and terminates when the participant fails to correctly score 3 out of 4 on a line. The participant is allowed one attempt to correct any error. The vision is scored as the line that the tested was completed on and 0.025 is subtracted for every error made on that line and on the preceding line.

Stereoacuity will be measured both by the pre-school Randot test, so as to be comparable with data from the PEDIG trials [16], and by the tablet-based Game Asteroid being developed by Newcastle University.

### Randomisation and codebreaking

Patients will be assigned sequentially, as each one consents to the clinical investigation, into the I-BiT Plus or control arms using the Simon and Pocock minimisation method (with an 80% chance of attempting to balance) in order to achieve a 1:1 ratio. The minimisation will be stratified by the recruitment centre, age (3.5 – < 6, 6 – < 9 and 9–12 years as the treatment response – visual acuity – is strongly age dependant over this age range for conventional treatment) and history of previous treatment (Yes/No).

The randomisation sequence was generated by the Derby Trials Unit.

The treatment allocation will be done using a web-based randomisation system and undertaken by the research orthoptist at the baseline visit.

It is not possible to mask the intervention and so there is no requirement for code breaking.

### Subsequent assessment

Participants will be assessed at weeks 3 and 6. Those who are randomised to the active treatment will also be assessed at week 10.

### Week-3 assessment

The week-3 visit will include visual acuity measurement, angle of strabismus and binocular vision status.

### Week-6 assessment

The week-6 visit will include visual acuity measurement, angle of strabismus and binocular vision status. Their quality of life will be checked using the CHU-9D and the CATQoL.

On completion of the 6 weeks, the equipment is retrieved from the participant’s home. The machine records how often and for how long it was used and this data is downloaded to a pen drive and separately analysed to determine compliance. The machine’s database is then cleaned prior to reuse.

### Week-10 assessment

The week-10 visit will include visual acuity measurement, angle of strabismus and binocular vision status.

Costs from an NHS and Personal Social Services perspective will be collected by combining recorded activity (caregiver questionnaires) with the appropriate unit cost. Intervention costs including I-BiT system and usual care, and any other relevant NHS resource utilisation within the duration of the trial will also be collected.

### Permitted and prohibited other interventions

Participants may not have patching or penalisation while participating in the trial. Otherwise treatment of other medical conditions is permitted.

### Masking

It is not possible to mask the intervention (as can be readily determined by the participant shutting their amblyopic eye). The clinical assessments are performed by an orthoptist masked to the intervention and the participant and parent instructed not to inform the assessing orthoptist. A record is kept of which orthoptist(s) are aware of the treatment allocation and is updated should the allocation get revealed to a particular individual.

### Outcomes

#### Primary outcome

There are two primary endpoints for the trial arms:

- Feasibility will be assessed by the number of hours of treatment delivered
- The primary clinical endpoint will be the change of visual acuity from baseline to week 6 post receipt of the I-BiT Plus or laptop between the two arms in each group

#### Secondary outcomes

The secondary outcomes for feasibility will include: recruitment rate and reasons for not taking part, treatment times and the proportion of patients completing a course of treatment (defined as 18 or more hours, which is 30 min per day, 6 days per week for 6 weeks), equipment breakages, retention rates and reasons for drop out, problems encountered with the randomisation process, completeness of outcome data and analysis of protocol deviations to determine rates and causes of non-compliance.

The secondary clinical out measures will include:

- Changes in visual acuity
- Changes in binocular status and includes stereoacuity
- Changes in angle of strabismus
- Quality of life outcomes

### Safety assessment

All adverse events will be recorded and categorised according to whether they are device related. Anticipated adverse events include headache, nausea, double vision, reverse amblyopia and photosensitive epilepsy.

The previous I-BiT trial did report two cases of diplopia were considered adverse device (I-BiT) [14] events which resolved on stopping treatment, while in the PEDIG trial the incidence was both low and considered similar in the dichoptic and the patched arms [17]. Reverse amblyopia and photosensitive epilepsy have not been reported.

Double vision may occur as part of the treatment process as breaking suppression may be necessary prior to developing fusion. It is left to the judgement of the site’s principle investigator as to whether it is appropriate for any participant who develops this, or any other adverse event, to remain in the trial or to be withdrawn.

Participants are covered by NHS indemnity.

### Sample size

This is a pilot study and, therefore, formal sample size estimations have not been performed.

Based on 20 treated patients per arm, the minimum importance difference of 0.1 logMAR in visual acuity between the I-BiT Plus arm and standard care arm can be observed at 5% significant level with 80% power if the standard deviation is 0.15 logMAR or less. We do not have an accurate estimate of the standard deviation and a known unknown is the compliance rate (both with the treatment and with the trial procedures). The standard deviation in visual acuity at 6 weeks will be estimated to inform sample size calculations for the future trial which will assess clinical effectiveness of the I-BiT™ system.

### Data management

The data is collected on paper case report forms and then sent to the Derby Clinical Trials Unit where the data is entered onto an electronic database according the Derby Clinical Trials Unit standard operating protocol.

Confidentiality is maintained by the case report forms and the datasets used for analysis containing trial number and initials only.

The final trial dataset will be managed by the Derby Clinical Trials Unit and access to the data will be only with permission of the trial management group prior to publication. Following completion of the trial, the data will archived and the possibility of depositing the data in a public archive will be explored.

### Management of trial documentation

Each site is given, at their site initiation visit, a trial site file with all the current trial documentation. Document updates, as they occur are sent to each site. In addition, there is an on-line document depository (Trello board) where all current documents are held and maintained and to which all sites have access to.

There is a project manager whose role includes ensuring that documentation is current and up to date with all relevant parties.

### Statistical methods

Statistical analysis will be performed for each of the three groups (anisometropic amblyopia, strabismic or mixed amblyopia, post strabismus surgery) separately. Intention to treat will be the primary analysis including all patients randomised regardless of the received treatment. The primary outcome of visual acuity change from baseline to 6 weeks will be analysed using analysis of covariance (ANCOVA) with baseline visual acuity, age and centre as the covariates for comparing the I-BiT Plus treatment vs the standard treatment.

We will examine the plausibility that primary outcome data is missing at random (MAR) and multiple imputation techniques will be used to handle missing values as appropriate. The estimated effect sizes and their 95% confidence intervals will be reported according to Consolidated Standards of Reporting Trials (CONSORT) guidelines [18]. The level of significance is defined at 0.05.

### Management and monitoring

No data monitoring committee has been appointed for this study due to the expected low incidence of adverse events and the low numbers in each arm. Routine data monitoring will be performed in accordance with the sponsor’s standard operating procedures. The trial is managed by Trial Management Group and with oversight from an independent Trial Steering Committee.

The Trial Management Committee has representation from the principle investigators from each of the trial sites, the project manager, the chief investigator, lead computer programmer, sponsor’s representative, representative from the Clinical Trials Unit and up to two academics from Nottingham University.

The Trial Steering Committee has an independent chair (consultant ophthalmologist from a different trust), a second independent ophthalmologist, a lay representative along with a sponsor’s representative, the trial statistician, the project manager and the chief investigator.

The sponsor has an obligation to ensure processes are followed but neither the sponsor nor the funders have any direct role in the study design, collection, management, analysis of the data, writing of the report of the decision to publish.

### Dissemination policy

The Trial Management Group, on behalf of the I-BiT Study Group will oversee publication of the trial data to the peer-reviewed literature.

### Ethical considerations

The study has received approval from both the Research Ethics Committee and Medicines and Health Regulatory Authority (MHRA) and will be conducted in accordance with the Declaration of Helsinki, ICH GCP and ISO14155.

## Discussion

This is a randomised controlled trial for the revised I-BiT™ system. There is now a body of evidence that dichoptic stimulation is an effective treatment for amblyopia [9] and this trial builds on previous work [13, 14, 19]. We have used the lessons from the previous studies to redesign the I-BiT system with the following improvements:

- Home-based system. Simple to use and also safe. For example, it will switch itself off if it is left idle for 15 min. The current system is designed to be used without supervision
- Range of games and videos (as opposed to one game and one video)
- Allow image off-setting for strabismic and mixed amblyopia
- Simple psychophysical tests incorporated
- Monitor activities undertaken and treatment time. It will not permit a treatment session to be longer than 1 h or more than 2 h in 1 day
- We also included a face verification system to help ensure that the person using the system is the intended participant

The major aim of this trial is as a feasibility study to see if all these features function as designed and at the same time get some indication of efficiency. The intention is to get sufficient information to allow a definitive study to be designed.

It should be noted that this approach is not without its problems. A major cause of treatment failure with patching is poor compliance [7, 20] but videogames must be engaging and can have their own compliance issues. In the PEDIG study, only 22% of participants complied with more than 75% of the prescribed treatment. The revised I-BiT has programmed up eight games in an attempt to ensure a reasonable choice and includes around 40 h of videos which are particularly targeted to the 3.5–6 years age range where they may be less inclined to the gaming options.

It is the issue of compliance that undermines any power calculation. We are just powered to detect a clinically significant difference but this is assuming good compliance. So it is likely that we will end up under powered, but we will have a measure of compliance which can be used for future calculations.

A second issue arises specifically for strabismic and mixed amblyopia. Only the fovea can support normal visual acuity and so it seems an important requirement that the dichoptic images are presented harmoniously. The revised I-BiT has this capability to off-set images by a required amount and it can measure the distance of the participant from the screen in real time to ensure that the angle of off-set can change with distance such that the degree of off-set as measured in prism dioptres is kept constant. This is a novel development.

We have chosen controls whose only treatment is the wearing of glasses, if indicated rather than patching. To use participants who are under-going patching or penalisation as controls would have made this a non-inferiority study and this requires an infeasibly large number of participants to generate a meaningfully powered study.

All participants, if they have significant refractive error, must have undergone at least 12 weeks of refractive adaptation. Refractive adaptation can occur over 30 weeks [5, 6] and it is standard practice to allow 18 weeks of refractive adaptation before considering patching or penalisation. By allowing enrolment at 12 weeks, it means that the controls can complete their participation in this study and be finished by 18 weeks and so this would not delay the start of conventional treatment, if indicated. It does mean that we anticipate some improvement in vision in the control arms.

The chosen method, described under trial procedures, of how to measure visual acuity was developed following a Delphi-like process involving all the paediatric ophthalmologists and orthoptists at the four participating trial centres. Although the tests are standard, it transpired that there was considerable variation on how these tests are applied in practice. It has been emphasised to all the trial centres the importance of consistency of making this measurement. It is a psychophysical measurement and such measurements are only valid if the subject is concentrating on the task and, particularly with children, this cannot be guaranteed. The variation in practice all revolved around how particular clinicians compensated for perceived failures in concentration by the subject. This is an area that would benefit from further research in its own right.

Patients with anisometropic amblyopia are required not to have had previous patching or penalisation whereas this is permissible for those with strabismic amblyopia. The reason is the age of presentation as most patients with strabismic amblyopia present at under the age of 3.5 years and this age is too young to consider the use of I-BiT. Patients with anisometropic amblyopia usually present at over the age of 3.5 years and so this makes I-BiT a feasible option.

Alongside these trials, data will be collected in order to perform a cost-utility analysis in the paediatric population which is particularly challenging due to the problem of accurately measuring utility in this age range [21]. Two questionnaires have been used to calculate Quality-adjusted Life Years (QALYs) have been the CHU-9D22 and the EQ-5D-Y23 with the former validated for the 7–11 years age range and the later from the age of 8 years. In the age range 6–7 years the CHU-9D performs better than the EQ-5D-Y and accordingly we have chosen it [22] and it also has a proxy form where a guardian can respond on their behalf. There is no instrument that is validated for children under the age of 6 years. However, it is noted that this area is problematic. We are also using a modified CAT-QoL which is a treatment-specific tool [23] as a disease-specific measure.

The revised I-BiT does have the ability to perform simple psychophysical testing. The participants in this study will be monitored clinically but the efficacy of this automated testing will be separately assessed as it may be possible, in the future, to monitor progress remotely and without the need for hospital visits.

This approach has the potential to transform the way patients with amblyopia are treated.

## Trial status

At the time of writing (April 2018), 34 patients have been recruited.

## Additional files

Additional file 1: Patient Information sheet written for adults (for the parents of participants). (DOCX 60 kb)

Additional file 2: Consent form for participation in the trial. (DOCX 55 kb)

Additional file 3: Assent form for particpants over the age of 6 years for participation in the trial. (DOCX 247 kb)

Additional file 4: List of members of the I-BiT study group (correct at time of manuscript submission). (DOCX 16 kb)

Additional file 5: Patient information sheet written for children. (DOCX 1496 kb)

Additional file 6: SPIRIT Checklist. (DOCX 30kb)

## Data Availability

The trial protocol and copies of the trial documentation is available from the corresponding author on request.
