# Supplementary material for: Clinical investigation plan for the use of interactive binocular treatment (I-BiT) for the management of anisometropic, strabismic and mixed amblyopia in children aged 3.5–12 years: a randomised controlled trial
Source: Trials. 2019 Jul 16;20:437. doi: 10.1186/s13063-019-3523-0 (PMC6636162; doi:10.1186/s13063-019-3523-0)
Supplement: Supplementary file 1 — Patient Information sheet written for adults (for the parents of participants). (DOCX 60 kb) [file 13063_2019_3523_MOESM1_ESM.docx]

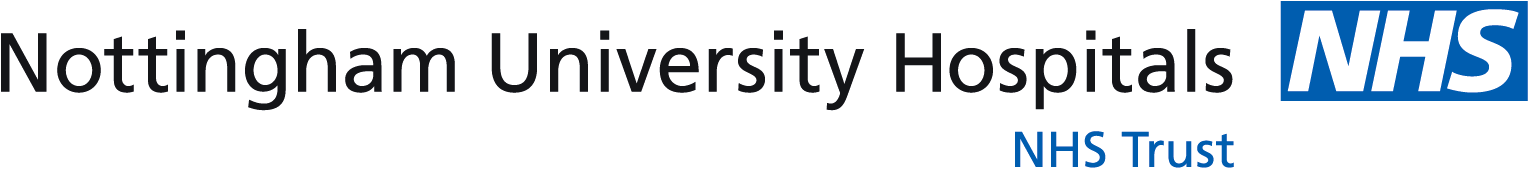


**INFORMATION FOR PARENTS / GUARDIANS**

Version 1.3 Dated 15^th^ December 2016

**Interactive Binocular Treatment (I-BiT) for the management of strabismic, anisometropic and mixed amblyopia**

**Randomised Controlled Trial**

We invite your child to take part in our research study. Before you decide, we would like you to understand why the research is being done and what it would involve for you and your child. **One of our team will go through the information sheet with you and answer any questions you have.**  Please talk to others about the study if you wish, and decide whether or not you wish your child to take part. Ask us if there is anything that is not clear.

**What is the purpose of the study?**

The usual method of treating amblyopia (lazy eye) is for the child to wear a patch over the good eye to make the lazy eye work. Although this can be a successful treatment, results from patching can be disappointing as some children dislike wearing a patch for several hours every day, sometimes over many months.

There is some evidence to suggest that glasses alone can improve vision, in some cases, for up to 30 weeks. In this trust, we usually allow 18 weeks of glasses only treatment, then start patching if vision is still reduced but for this study patients must have completed a minimum of 12 weeks with glasses. We would like to compare the approach of more weeks of glasses only treatment with the new treatment below.

We want to test a new method of treating amblyopia (lazy eye), which is a computer-based treatment and uses virtual reality technology. It is called the I-BiT system (I-BiT means Interactive Binocular Treatment). When the system is being used for treatment, the child has to wear special glasses which makes them use their lazy eye in order to play computer games or watch DVDs. The system is enjoyable for children to use and is well tolerated by children who are keen to watch the DVDs and play the computer games.

Participation in the study will require you to be prepared to have the I-BiT system (which is based on a personal computer with a high definition screen) delivered to and set up in your home for your child to use most days as advised, for 6 weeks. You will also need to attend the hospital for a baseline assessment before starting the treatment, part way through the treatment (3 weeks into treatment), at the end of the treatment (6 weeks after starting), and 4 weeks later for a standard full assessment (a total of 4 visits over approx. 10 weeks).

**Why has my child been invited?**

Your child has been chosen because they have the type of amblyopia (lazy eye) we think should be treated with our new system. Also they are the right age for treatment.

**Do we have to take part?**

It is up to you to decide if you wish your child to join the study. We will describe the study and go through this information sheet with you. If you agree for your child to take part, we will then ask you to sign a consent form. You are free to withdraw at any time, without giving a reason. This would not affect the standard of care your child will receive.

**What do we have to do?**

The system we are testing uses computer games and DVDs which are viewed through a special pair of 3D glasses. The system has been specially prepared so that most of the picture is seen by the amblyopic (lazy eye) and not the good eye.

If your child took part in this randomised controlled study, he or she may be asked to play computer games and/or watch a DVD for a minimum of 30 minutes on most days, wearing special glasses.

They would have to have their eyes examined

- before the study began (week ‘zero’)
- during their treatment (at three weeks)
- at the end of their treatment (at six weeks)
- 4 weeks after they finished their treatment (at week 10)

As part of the research process, we are required to collect information about how the trial affects the participant’s quality of life. As the participants in this study are children, their parent of carer will be asked to answer the questions on the child’s behalf (children can be asked for their opinion but the parent will fill in the questionnaire). We will ask you/your child to fill in one questionnaire at week ‘zero’ and two questionnaires at week 6. We will also ask you some questions about your need to access other healthcare services e.g. GP or A&E visits. As your child’s treatment is delivered at home, we will need to ensure that the treatment is only delivered to your child. The IBiT System will record the distance between their eyes and the size of their eyes creating an image which will be stored and used to improve face confirmation software in the future. We will not be able to identify your child from this image.

**What is a randomised controlled study?**

Randomised controlled studies are used when we don’t know which the best way of treating patients is. To find out the best way, we need to compare different treatments. We put people into groups and give each group a different treatment. To try to make sure the groups are the same to start with, each patient is put into a group by chance (which is like “throwing a dice”). This is called randomisation. The patient cannot choose which treatment they receive. The results are compared at the end of the study to see if there is any difference between the treatments.

The randomisation process will allocate your child to either treatment or control:

- **Treatment**: Children will watch DVDs and play specially prepared games using the I-BiT system with the 3D glasses.
- **Control**: Children will continue to wear their glasses (if glasses have been prescribed) for a further 6 weeks and play the same I-BiT games and watch DVDs (placebo games/DVDs, **not** the specially prepared version) on a standard laptop with **no** 3D glasses.

It is likely that the vision will improve in both the treatment and the control groups as wearing glasses alone will result in visual improvement for up to 30 weeks from the starting of wearing them. The trial is to see if the playing of games or watching DVDs designed to stimulate the lazy eye results in further improvement in the treatment group.

To try to make sure that the results are as good as possible, a different Orthoptist who doesn’t know what treatment your child is receiving will test your child’s eyes. The research Orthoptist who tells you about the study and helps you and your child during the study, will not know the results from your child’s eye tests during the study. This means that all children can be looked after in the same way no matter what treatment they receive and what the results of their eye tests are.

**How does this treatment differ from the usual treatment?**

The usual method of treating amblyopia (lazy eye) is to wear a patch over the good eye, every day for up to six hours a day. This treatment may last many months. If your child had this patching treatment, they would have to attend the Orthoptic clinic every six to eight weeks to have their vision assessed.

The treatment in this study is given daily for 6 weeks. Your child would have to play computer games and watch films for a minimum of 30 minutes (maximum of 2 hours) per day on average. They would also have to come to hospital to have their eyes tested during the treatment. They would then have to come back to the clinic 4 weeks after they finished their treatment (a total of 4 visits over approx. 10 weeks).

If your child did take part in the study, you would have to come to the clinic more often than if they were having conventional treatment. To cover the cost of these extra visits during the study, travel expenses would be paid, up to a maximum of £15 per visit, at each **extra** visit during the study (i.e. out of 4 visits during the study, 3 of them would be extra to normal).

| **I-Bit Study** | **Usual Treatment** |
| --- | --- |
| - Attend to find out if they are able to take part in the study, have vision tested and usual Orthoptic assessment, sign consent form and fill in questionnaires. - Have I-BiT or control system at home. - Vision test and Orthoptic assessment will be performed again at week 3 and 6. - Treatment stopped after 6 weeks and questionnaires filled in again. I-BiT/ control system returned to hospital. - Vision/Orthoptic assessment at week 10 (4 weeks after treatment stopped). - Continue with usual treatment if necessary after week 10 assessment. - Total of **4 trial visits over 10 weeks**, followed by usual visits as necessary. | - Attend to have Vision/Orthoptic assessment and start patching treatment. - Prescribed eye patch over the good eye for up to 6 hours a day as well as any glasses needed. - Regular Orthoptic assessments every 6-8 weeks until treatment is stopped. - Total number of visits depends on how vision improves but to compare with trial visits; usual treatment requires **4 visits over 24 weeks**. |
|  |  |
|  |  |
|  |  |

**Will the delay in treatment affect the final vision?**

No, if your child receives the I-BiT treatment we are hopeful that the vision will improve. If the vision remains reduced after six weeks, your child can continue with the standard patching treatment. If your child is randomised into the control group to continue glasses only treatment and play placebo games, a short gap of six weeks without additional treatment is too short a period of time to affect your child’s final vision in their lazy eye. They can start the standard patching treatment straight after the study time.

**What are the alternative treatments for Amblyopia (lazy eye?)**

In addition to patching treatment already described, other treatment methods include blurring the vision in the good eye by using eye drops. Sometimes using eye drops in this way is chosen as a first treatment instead of patches, but your Orthoptist will advise you of the best treatment for your child’s specific eye condition. With this treatment, visits are usually every 4 weeks during treatment.

**What are the possible disadvantages and risks of taking part?**

If the I-BiT treatment system does not improve vision to a level that we would like, then at the end of the study your child can have patching or other appropriate treatment. We do not expect the delay caused by taking part in the study to have any bad effects.

**What are the side effects of taking part?**

We do not expect our system to have any side effects. We know that in some virtual reality systems you can feel that you are moving and so you can develop travel sickness, but we have not had any problems like this so far. There is a very small chance that some children, who have a squint, may develop double vision after using our system. Patients with amblyopia and squints do sometimes develop double vision whilst under normal care. This is usually transient (temporary) and it is very rare for it to give rise to long term problems. If it did happen as a result of the trial, we would expect that it would be for a few days only. The system can flag up any concerning results to reduce this risk, and patching can also, very rarely, have the same effect.

**What are the possible benefits of taking part?**

The trial is designed to tell us how well each treatment works, so we don’t know for sure whether any of the treatments will be successful although small studies have given some positive signals. However, there is the chance that your child could benefit as their vision could improve without them having to be treated with patching. They may also have some fun playing the computer games and watching the DVDs.

By taking part in the trial, they will also help us to understand this condition better so that we can help improve children’s vision and prevent this common cause of childhood blindness in one eye.

**What if new information becomes available?**

Sometimes in the course of a research project new information about the disease or treatment becomes available. In our study, however, we think that this is very unlikely as other researchers are not looking for new treatments for lazy eye. If it does happen though, we will tell you about it and discuss with you whether the research project should continue or whether you might want to withdraw from the study.

**What will happen when the research study stops?**

Your child’s vision will be assessed at the end of the trial. Your child will then go back to the normal Orthoptic department where their future treatment would be discussed with you. Participation in this study has no impact on future treatment options.

**What if something goes wrong?**

We don’t think there is any danger involved with our study. If someone is harmed as a result of the study, there are no special arrangements for compensation. However, the normal NHS complaints mechanisms will still be available for you. If you have a concern about any aspects of this study, you should ask to speak to one of the research Orthoptists involved who will do their best to answer your questions (Telephone no. 0115 970 9750). If you remain unhappy and wish to complain formally you can do this. Details can be obtained from the Patient Advice & Liaison Service - PALS (Telephone number, freephone: 0800 183 0204)

**Will my child taking part in this study be kept confidential?**

All information which is collected about your child during the course of the research is strictly confidential and stored securely. If your child joins the study some parts of their records and data collected will be looked at by authorised persons. Their GP will be notified that they are in the study, but not the treatment that they are receiving or their individual results.

**What will happen to the results of the research study?**

The results of the study will be published in medical and scientific journals and presented and discussed at scientific conferences and meetings of doctors and researchers. We will also publish a summary of the results on the trial website which you will be able to see, and invite you to a meeting to hear the results once they are available. Your child cannot be identified individually at any point.

**Who is organising and funding the research?**

The research is funded by the NHS National Institute for Health Research (NIHR). Your child’s doctor will not be paid for including them in the study. The project is organised by the Orthoptic department and eye doctors in the participating centres. The development of the I-BiT system and virtual reality technology takes place at the University of Nottingham and also the University of Hull.

**Who has reviewed the study?**

All research in the NHS is looked at by an independent group of people, called a Research Ethics Committee, to protect your interests, rights and wellbeing. This study has been reviewed and given a favourable opinion by Yorkshire and Humber – Leeds West Research Ethics Committee.

**Contact for further information**:

*Rebecca Brown*

*I-BiT Project Manager/Orthoptist*

*Orthoptic Department*

*Queen’s Medical centre, Nottingham*

*Tel:* ***07812 270155 (please leave a message)***

*Or 0115 9709750*

*Email:* [*ibit@nuh.nhs.uk*](mailto:ibit@nuh.nhs.uk)

*Website:* [*www.lazy-i-bit.co.uk*](http://www.lazy-i-bit.co.uk)

**Thank you for taking time to read this information**

***The parents/guardian will be given a copy of this information sheet and a signed consent form to keep.***
