# Supplementary material for: Clinical investigation plan for the use of interactive binocular treatment (I-BiT) for the management of anisometropic, strabismic and mixed amblyopia in children aged 3.5–12 years: a randomised controlled trial
Source: Trials. 2019 Jul 16;20:437. doi: 10.1186/s13063-019-3523-0 (PMC6636162; doi:10.1186/s13063-019-3523-0)
Supplement: Supplementary file 2 — Consent form for participation in the trial. (DOCX 55 kb) [file 13063_2019_3523_MOESM2_ESM.docx]

**PARENTS / GUARDIANS CONSENT FORM**

Version 1.3 Dated 15^th^ December 2016

**Interactive Binocular Treatment (I-BiT) for the management of Strabismic, Anisometropic and Mixed Amblyopia.**

**Randomised Controlled Trial**

**Centre number:**

**Study Number:**

**Patient Identification Number for this trial:**

**Name of Researcher: Please initial box**

I confirm that I have read and understand the information sheet dated.................... (version............) for the above study. I have had the opportunity to consider the information, ask questions and have had these answered satisfactorily.

I agree to complete, on behalf of my child, the quality of life questionnaires at weeks 0 and 6 and questions on use of health care services.

I understand that my child’s participation is voluntary and that I am free to withdraw my child at any time without giving any reason, without my child’s medical care or legal rights being affected.

I understand that relevant sections of my child’s medical notes and data collected during the study may be looked at by individuals from the I-BiT team, from regulatory authorities or from the NHS Trust, where it is relevant to my child’s taking part in this research. I give permission for these individuals to have access to my child’s records.

I understand that in order to ensure that the treatment is only delivered to my child, the I-BiT system will record the distance between their eyes and the size of the eyes, creating an unidentifiable image which will be stored and used to improve face confirmation software in the future.

I agree to my son’s/daughter’s GP being informed of my child’s participation in the study.

I agree for my son/daughter to take part in the above study

Name of Child…………………………………………………………………………………………..

Name of Parent / Guardian……………………………………………………………………………

Signed……………………………………………………………. Date…………………..................

Name of person taking consent……………………………………………. ………………………..

Signed……………………………………………………………. Date………………….. ………….

1 copy for participant, 1 copy for researcher, 1 copy for the medical notes
