# Supplementary material for: Clinical investigation plan for the use of interactive binocular treatment (I-BiT) for the management of anisometropic, strabismic and mixed amblyopia in children aged 3.5–12 years: a randomised controlled trial
Source: Trials. 2019 Jul 16;20:437. doi: 10.1186/s13063-019-3523-0 (PMC6636162; doi:10.1186/s13063-019-3523-0)
Supplement: Supplementary file 3 — Assent form for particpants over the age of 6 years for participation in the trial. (DOCX 247 kb) [file 13063_2019_3523_MOESM3_ESM.docx]

ASSENT FORM

A new treatment for Lazy Eyes called I-BiT (Trial)

AGED 6 to 12

Version 1.2 03 August 2016

IRAS no. 198518

**Young person to circle all they agree with please:**

Have you read (or had read to you) about this project? Yes No

Has somebody else explained this project to you? Yes No

Do you understand what this project is about? Yes No

Have you asked all the questions you want? Yes No

Have you had your questions answered in a way you understand? Yes No

Do you understand it’s OK to stop taking part at any time? Yes No

Are you happy to take part?
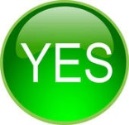

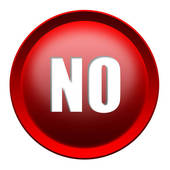


Name of child or young person (PRINT)………………… Date of Birth...........................

Name of person obtaining consent (PRINT)……………………… Signature………………… Date…………………

**Optional**

Name of mother/father/Guardian(s) (PRINT)…………………………………………………………………..

Signature…………………………………………….. Date…………………………...
