# Supplementary material for: Clinical investigation plan for the use of interactive binocular treatment (I-BiT) for the management of anisometropic, strabismic and mixed amblyopia in children aged 3.5–12 years: a randomised controlled trial
Source: Trials. 2019 Jul 16;20:437. doi: 10.1186/s13063-019-3523-0 (PMC6636162; doi:10.1186/s13063-019-3523-0)
Supplement: Supplementary file 4 — List of members of the I-BiT study group (correct at time of manuscript submission). (DOCX 16 kb) [file 13063_2019_3523_MOESM4_ESM.docx]

| Name | Institution | E-mail | Role |
| --- | --- | --- | --- |
| Adams, Gill | Moorfields Eye Hospital (MEH) | gill.adams@moorfields.nhs.uk | Principal investigator at MEH |
| Ash, Isabel M | Nottingham University Hospital Trust NUH) | Isabel.ash@nuh.nhs.uk | Head of orthoptics at NUH |
| Attwood, Alison | Royal Stoke University Hospital (RSUH) | alison.attwood@uhnm.nhs.uk | Orthoptist at RSUH |
| Blanchfield, Peter | Nottingham University | pszpxb@exmail.nottingham.ac.uk | Lead Programmer |
| Bradley, Matthew | Nottingham University | matthew.t.bradley@gmail.com | Computer Programmer |
| Brown, Rebecca | NUH | [Rebecca.brown4@nuh.nhs.uk](mailto:Rebecca.brown4@nuh.nhs.uk) | Project Manager and orthoptist |
| Chester, Victoria | Derby Trials Unit | victoria.chester@nhs.net | Triallist |
| Clements, Fiona | RSUH | fiona.clements@uhnm.nhs.uk | Orthoptist at RSUH |
| Eastgate, Richard M | Nottingham University | epzrme@exmail.nottingham.ac.uk | Co-Investigator |
| Fakis, Apostolos | Derby Trials Unit | apostolos.fakis@nhs.net | Trial Statistician |
| Foss, Alexander J.E. | NUH | Alexander.foss@nottingham.ac.uk | Chief investigator |
| Harrad, Richard | University Hospitals, Bristol | r.a.harrad@bristol.ac.uk | Chair of trial steering group |
| Harris, Roy | NUH | Royharris53@virginmedia.com | Advisor on intellectual property and co-applicant |
| Jones, Ruth | RSUH | ruth.jonesres@uhnm.nhs.uk | Research nurse at RSUH |
| Joseph, Annie | RSUH | Annie.joseph@uhns.nhs.uk | Principal investigator at RSUH |
| Kusuman, Keerthy | Nottingham University | pszkk@exmail.nottingham.ac.uk | Computer programmer |
| Lavalle, Peter | Nottingham University | peter.lavalle@nottingham.ac.uk | Computer programmer |
| Matthews, Karen | RSUH | karen.matthews@uhnm.nhs.uk | Orthoptist at RSUH |
| Mischo, Meike | Sensomotoric Instruments | Meike.mischo@smi.de | Provider of eye-trackers |
| McGraw, Paul | Nottingham University | lpzpvm@exmail.nottingham.ac.uk | Visual psychologist and co-applicant |
| Osbourne, Dan | Southampton General Hospital | daniel.osborne@uhs.nhs.uk | Research orthoptist |
| Patel, Dipesh, E | MEH | dipesh.patel@moorfields.nhs.uk | Research clinician |
| Purdy, Jon | Hull University | j.h.purdy@hull.ac.uk | Computer programmer and co-applicant |
| Ranger, Megan | Southampton General Hospital | megan.ranger@uhs.nhs.uk | Research orthoptist |
| Shaw, John | Lay representative | sesjws@btinternet.com | Member of trial steering group |
| Stimpson, Ed | NUH | edward.stimpson@nuh.nhs.uk | Contracts |
| Thomas, Shery | NUH | shery.thomas@nuh.nhs.uk | Principal investigator at Nottingham site |
| Vivian, Anthony | Addenbrookes hospital | vivianeye@aol.com | Advisor |
| West, Stephanie | Southampton General Hospital | stephanie.west@uhs.nhs.uk | Principal investigator at Southamptom |
| Worrall, Laura | RSUH | laura.worrall@uhnm.nhs.uk | Research orthoptist |
| Wragg, Andrew | NUH | andrew.wragg@nuh.nhs.uk | Patient and public involvement co-ordinator |
