# Supplementary material for: Clinical investigation plan for the use of interactive binocular treatment (I-BiT) for the management of anisometropic, strabismic and mixed amblyopia in children aged 3.5–12 years: a randomised controlled trial
Source: Trials. 2019 Jul 16;20:437. doi: 10.1186/s13063-019-3523-0 (PMC6636162; doi:10.1186/s13063-019-3523-0)
Supplement: Supplementary file 5 — Patient information sheet written for children. (DOCX 1496 kb) [file 13063_2019_3523_MOESM5_ESM.docx]

**A story/information leaflet for children having**

**A new treatment for Lazy Eyes called I-BiT (Trial)**

Version 1.2 Dated 03^rd^ August 2016


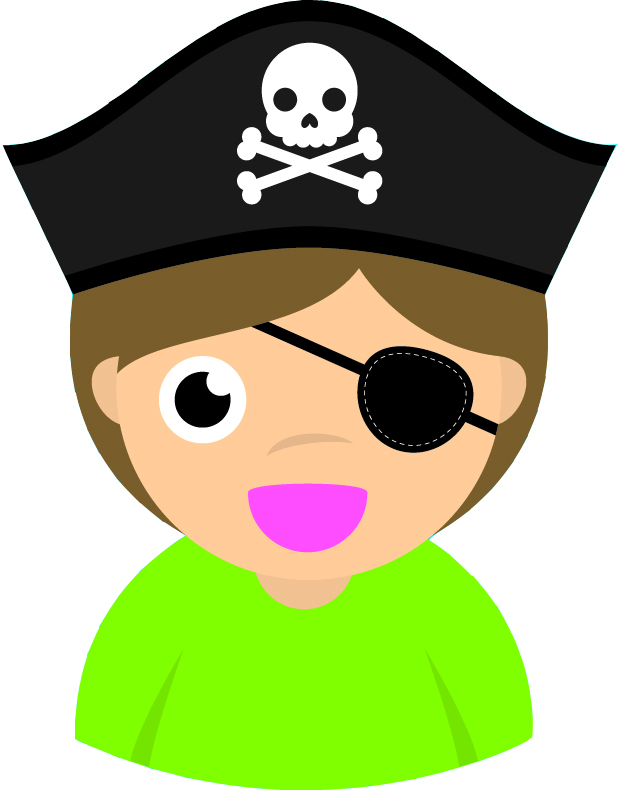
Your eyes don’t work the way we would like them to. One eye sees very well and looks at everything, but the other eye is lazy and does not work so well. This can make you less good at doing some things such as some games and sports. We would like to fix that.

Some children have a patch put over their good eye to make their lazy eye do some of the work. Some children like this as they can play at being a pirate, but some children don’t like it so much.

So, we are trying to find another way to help children’s eyes work better. We have designed a special computer where you wear special glasses and you can either play computer games or watch programmes/films as you wish.


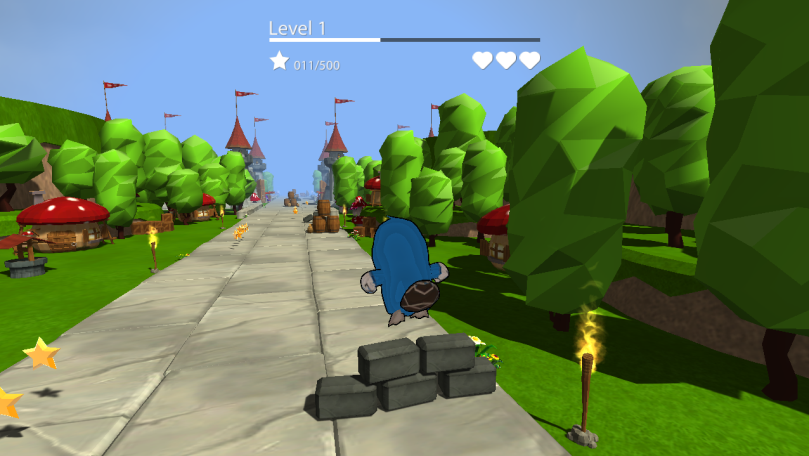
In this study, you will borrow a computer from the hospital and play on the games and watch films or programmes for six weeks. Some children will have a normal computer and others will have the special computer; the games and films will be the same on both. We will choose who gets which computer.

You can take the computer home and play on it. You will be asked to play on it for at least half an hour on most days.


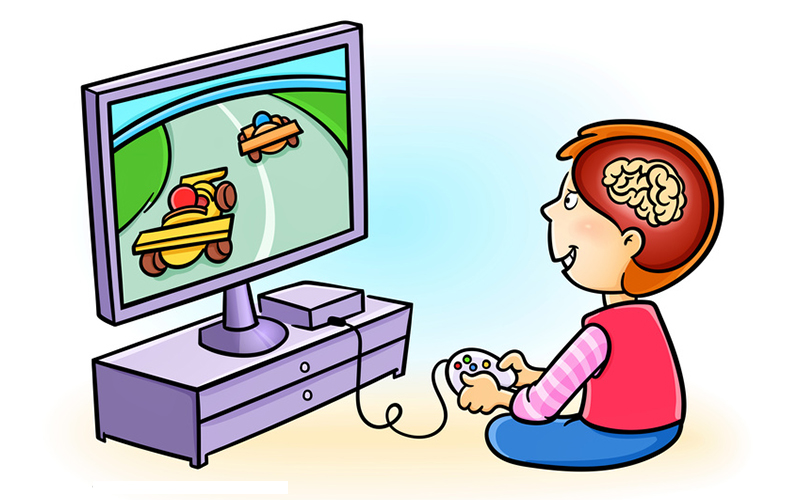


You get special eye tests to check how your eyes are on some days. The person who will test your eyes isn’t supposed to know which computer that you have had, so you need to keep this a secret.

We have good reason to think that the special computer will help the lazy eye see better but this is not proven and that is why we are asking for your help.

Your mum, dad, guardian or special eye nurse can explain more about this to you if you want. Don’t be afraid to ask questions.
